# Development and Preliminary Evaluation of a Serum Blocking ELISA for the Detection of PEDV Antibodies and Assessment of Its Association with Neutralizing Antibody Titers

**DOI:** 10.3390/ani16213465

**Published:** 2026-11-02

**Authors:** Fangshan Wang, Yawen Wang, Jing Ren, Xue Zhao, Zelin Liu, Tairan Sun, Lihua Cheng, Jinhai Huang, Qinye Song

**Affiliations:** 1 College of Veterinary Medicine, Hebei Agricultural University & Veterinary Biological Technology Innovation Center of Hebei Province, Baoding 071000, China; 2 Baoding Animal Disease Prevention and Control Center, Baoding 071051, China; 3 Raoyang County Centre for the Prevention and Control of Animal Diseases, Hengshui 053900, China; 4 School of Life Sciences, Tianjin University, Tianjin 300072, China

**Keywords:** porcine epidemic diarrhea virus, S1 protein, blocking ELISA, serological monitoring, neutralizing antibodies

## Abstract

Porcine epidemic diarrhea virus (PEDV), the etiological agent of porcine epidemic diarrhea (PED), remains a major threat to swine production worldwide. The genetic variation and the co-circulation of multiple genotypes of this virus may compromise vaccine-induced protection, creating an urgent need for practical serological tools for herd-level monitoring. A blocking enzyme-linked immunosorbent assay (blocking ELISA) was developed using a recombinant truncated PEDV S1 protein as the coating antigen and a horseradish peroxidase (HRP)-labeled anti-PEDV S1 monoclonal antibody as the competing detector. No cross-reactivity was detected with the tested antisera against porcine reproductive and respiratory syndrome virus (PRRSV), pseudorabies virus (PRV), classical swine fever virus (CSFV), or porcine circovirus type 2 (PCV2). The intra-assay and inter-assay coefficients of variation were 3.02% and 3.76%, respectively. Receiver operating characteristic analysis yielded an optimal cutoff of 31.76%, with a sensitivity of 98% and a specificity of 99%. Among 33 clinical serum samples, blocking-ELISA classifications were fully concordant with infected-cell indirect immunofluorescence assay (IFA) results (33/33, 100%), which were interpreted as a measure of general PEDV seroreactivity. Comparison with virus neutralization testing of 98 sera showed category-specific concordance rates of 81.25%, 90.91%, and 100% for neutralizing antibody titers of 1:64, 1:128, and ≥1:256, respectively. All sera in the ≤1:16 category were negative by blocking ELISA. A continuous correlation analysis between the blocking percentage and neutralizing antibody titer was performed (ρ = 0.484, 95% CI 0.315–0.623, *p* = 5.1 × 10^−7^; *n* = 97). Compared to traditional neutralization tests, this method features simpler operation, faster detection, and higher efficiency, rendering it suitable for large-scale monitoring of vaccine efficacy in swine herds and offering robust technical support for precise evaluation of PEDV immunity.

## Simple Summary

Porcine epidemic diarrhea causes severe diarrhea and death in newborn piglets. Measuring antibodies in breeding herds may help veterinarians monitor responses to vaccination and identify animals that need further assessment. However, laboratory tests that measure the ability of antibodies to prevent infection are time-consuming. We developed a blood test using a selected part of the viral surface protein and an antibody that recognizes this protein. The test detected antibodies against porcine epidemic diarrhea virus without reacting with the tested antibodies against several other common pig viruses. It also produced consistent results across repeated measurements. Comparisons with cell-based tests showed that the assay was particularly effective at identifying blood samples with higher levels of virus-neutralizing antibodies. These findings support its potential use for routine antibody monitoring in pig herds. Further studies using samples from sows and their piglets are needed to determine whether the test can predict protection passed from mothers to offspring.

## 1. Introduction

Porcine epidemic diarrhea (PED), caused by porcine epidemic diarrhea virus (PEDV), remains one of the most consequential enteric diseases affecting neonatal piglets and the global swine industry. PEDV is classified within the genus *Alphacoronavirus* of the family *Coronaviridae*, and infection is characterized by acute diarrhea, vomiting, severe dehydration, and high neonatal mortality [1,2]. Since highly pathogenic PEDV variants, Gene group 2 (G2), emerged in China around 2010, the virus has continued to circulate through contaminated feces, equipment, environments, and mammary gland secretions. In severe outbreaks, mortality among suckling piglets may reach 80–100% [3]. Control is further complicated for the high mutation rate of the PEDV genome, particularly in the spike (S) gene, which may diminish the protective efficacy of classical vaccines and underscores the need for reliable tools for diagnosis, immune-status monitoring, and vaccine evaluation [4,5]. Accordingly, early diagnosis and sustained herd-level surveillance are important for interrupting transmission and maintaining stable pig production [6,7,8].

S protein is a surface-exposed glycoprotein that is central to PEDV entry and host-cell tropism. The two functional subunits (S1 and S2) of S protein perform distinct but coordinated roles: S1 at the N-terminus is mainly responsible for receptor attachment, whereas S2 at the C-terminus drives membrane fusion following proteolytic activation. In cell culture, trypsin does not act as a receptor-binding factor. Instead, it facilitates viral entry by cleaving receptor-bound S protein and thereby promoting membrane fusion [9]. In addition to mediating entry, S is the principal target of neutralizing antibodies. Genetic variation in this protein, especially within S1, is closely associated with antigenic drift and altered immune protection [10,11]. Recent epidemiological evidence indicates that G2c/GIIc-like strains have become predominant in China, and mutations in their S genes may affect major antigenic epitopes [12]. These biological and epidemiological features make the neutralizing and immunodominant regions of S, including COE, SS2, SS6, and other S-region epitopes, important targets for both vaccine evaluation and serological assay development [13,14,15].

Against this background, serological testing is widely used to assess PEDV exposure, vaccine-induced antibody responses, and maternal antibody status. ELISA-based platforms are particularly suitable for routine herd-level monitoring because they are economical, rapid, and readily adaptable to high-throughput testing [16]. Monoclonal antibody-based competitive ELISA and nanobody-based blocking ELISA detect PEDV antibodies through competition between serum immunoglobulins and a defined antibody reagent, an approach that can enhance analytical specificity [16,17]. Indirect ELISA based on full-length or truncated S1 antigens have also been applied to quantify anti-PEDV IgA responses in serum, colostrum, and milk [18]. A further application is prediction of maternally derived passive protection: maternal serum antibody measurements could provide an accessible indicator of the immune response supplying antibodies to offspring, provided that their relationship to lactogenic transfer and piglet outcomes is established. Published associations between sow serum antibodies and litter survival, together with maternal-vaccination and passive-transfer experiments, support this premise [4,19,20]. We therefore developed an S1 blocking ELISA and evaluated its preliminary serum performance through repeatability, cross-reactivity, IFA comparison and the distribution of ELISA results across neutralization-titer categories. We also examine the biological rationale for its potential use as a predictor of passive protection, while treating direct maternal transfer and outcome prediction as subsequent validation objectives.

## 2. Materials and Methods

### 2.1. Antigens, Reagents, and Antibodies

Mouse-derived PEDV S1 protein monoclonal antibody was prepared and stored in the Infectious Disease Laboratory of Hebei Agricultural University. This monoclonal antibody was generated via hybridoma cell fusion technology and is of IgG2bκ isotype, targeting the ^55^IGENQGVNSTWYCAGRHPTAS^57^ epitope on the PEDV-S1 protein, with an ascites antibody titer of 1 × 10^6^. A panel of 90 serum samples was obtained from PEDV-vaccinated pig farms. PRRSV-, CSFV-, PRV-, and PCV2-specific antisera were also prepared in our laboratory, standardized, and stored under cryopreservation conditions.

TRIzol reagent, dNTPs, Taq DNA polymerase, and a centrifugal column-based DNA gel extraction kit were purchased from Tiangen Biotech (Beijing, China) Co., Ltd. Reverse transcriptase was obtained from Promega Corporation (Madison, WI, USA), and EcoR I and Xho I restriction endonucleases were purchased from TaKaRa Bio (Dalian, China) Co., Ltd. The Ni-Agarose His-tag protein purification kit was obtained from CWBIO (Beijing, China), and the protein quantification kit was purchased from Nanjing Jiancheng Bioengineering Institute (Nanjing, China). HRP-conjugated goat anti-pig or anti-mouse IgG was purchased from Solarbio (Beijing, China), and the rapid HRP-labeling kit was supplied by Beijing BioLong Immunotechnology Co., Ltd. (Beijing, China). Porcine antisera against PEDV, PCV2, PRRSV, CSFV, PRV, TGEV, and RVA, together with mouse anti-PEDV-positive and -negative sera, were prepared and stored in our laboratory.

### 2.2. Cells and Expression Vector

Competent *E. coli* DH5α and BL21 cells, the prokaryotic expression vector pET28a (+), as well as Vero-81 cells were maintained in our laboratory.

### 2.3. Preparation and Characterization of Recombinant S1 Protein

A 945-bp PEDV S1 coding fragment was amplified using primers U1 (5′-ACTGAATTCATGGTACTGGGCGGTTATCTA-3′) and L1 (5′-ATTCTCGAGTTAGGCTAAGTGAGGATCTGA-3′) by PCR, cloned into pET28a(+) via *EcoR* I and *Xho* I sites, and verified by restriction digestion and Sanger sequencing. The successfully constructed expression vector was designated pET28a-S1.

Next, the recombinant pET28a-S1 construct was expressed in *E. coli* BL21 under induction with 0.5 mmol/L IPTG at 35 °C for 5 h. The His-tagged antigen was purified using Ni-Agarose affinity purification. Protein expression, the protein purified fraction and the distribution of recombinant protein between the pellet and supernatant fractions were assessed by SDS-PAGE, with the empty-vector strain as the expression control. Recognition of the recombinant antigen was examined by Western blotting using mouse anti-PEDV S1 serum (1:320) and HRP-conjugated goat anti-mouse IgG (1:2000). Subsequently, the S1 protein was purified using a His-tagged protein purification kit (Beyotime, Shanghai China).

### 2.4. Establishment and Evaluation of the PEDV Blocking ELISA

Assay development included optimization of HRP-labeled monoclonal antibody dilution, the antigen coating concentration, test-serum dilution, blocking buffer, antibody diluent, coating time, blocking time, and antibody incubation time. The cutoff was determined by ROC analysis. Repeatability, cross-reactivity within the tested antiserum panel, detection across serial serum dilutions, and categorical agreement with IFA and virus neutralization results were subsequently evaluated using the serum panels described below.

HRP labeling of the PEDV S1 monoclonal antibody. The anti-PEDV S1 monoclonal antibody was labeled with a commercial rapid HRP-labeling kit according to the manufacturer’s instructions. Purified antibody (500 μL) was combined with 50 μL of labeling buffer and mixed gently to avoid foaming. HRP solution (50 μL) was added, and the mixture was pipetted gently until homogeneous and incubated for 3 h at room temperature in the dark. A 60-μL of 2.5 mg/mL termination solution was added to the labeling reaction, mixed thoroughly, and incubated for a further 1 h at room temperature in the dark. Finally, 660 μL of preservation buffer was added, and the labeled antibody was mixed gently and stored at −20 °C until use.

Determination of the working dilution of the HRP-labeled antibody. PEDV S1 protein was diluted to 10 μg/mL in 0.05 M carbonate buffer (pH 9.6), added to ELISA plates at 100 μL/well, and incubated at 4 °C for 12 h. After three washes with PBST, the plates were blocked with 5% newborn bovine serum in PBST at 37 °C for 1 h. The HRP-labeled anti-PEDV S1 monoclonal antibody was diluted in 1% BSA-PBS to 1:100, 1:200, 1:400, 1:800, 1:1600, 1:3200, 1:6400, or 1:12800 and added at 100 μL/well. The corresponding unlabeled ascites and blank wells without antigen coating were also set up served as the ascites control and blank control, respectively. After incubation at 37 °C for 1 h and washing, a 50-μL volume of each of (3,3′,5,5′-tetramethylbenzidine, TMB) substrate solutions A and B was added per well. Color was developed at 37 °C for 20 min, and reactions were stopped with 50 μL of 2 M H_2_SO_4_. OD_450_ values were used to select the working dilution of the labeled antibody.

Optimization of antigen coating concentration and HRP-labeled monoclonal antibody dilution ratio. PEDV S1 protein was diluted in carbonate buffer (pH 9.6) to 0.5, 1, 2, 4, or 8 μg/mL and dispensed into 96-well ELISA plates at 100 μL/well. Plates were incubated at 37 °C for 1 h and then at 4 °C overnight. After washing, the plates were blocked with 5% newborn bovine serum in PBST for 1 h at 37 °C. Anti-PEDV-positive and -negative sera diluted 1:100 were added at 100 μL/well and incubated for 1 h at 37 °C. After washing, HRP-labeled anti-PEDV S1 monoclonal antibody diluted 1:100, 1:200, 1:400, or 1:800 in 1% BSA-PBS was added at 100 μL/well and incubated for 45 min at 37 °C. TMB substrate was added after washing, and the reaction was stopped with 2 M H_2_SO_4_. The antigen concentration and HRP-labeled monoclonal antibody dilution ratio were selected according to PI values.

Determination of the dilution of test serum. Plates were prepared using the selected coating concentration and incubation conditions. After blocking, anti-PEDV-positive and -negative sera were diluted 1:50, 1:100, 1:200, or 1:400 in 5% newborn bovine serum-PBST and added at 100 μL/well. Following incubation at 37 °C for 1 h and washing, the HRP-labeled anti-PEDV S1 monoclonal antibody, diluted 1:400 in 1% BSA-PBS, was added and incubated at 37 °C for 45 min. Reactions were developed with TMB and stopped with 2 M H_2_SO_4_. The serum dilution ratio was determined based on the PI value and separation performance between the positive and negative sera.

Screening of blocking buffer and HRP-labeled antibody diluent. ELISA plates were coated under the selected conditions and blocked with either 5% newborn bovine serum (NBS) in PBST or 1% BSA-PBST for 1 h at 37 °C. PEDV-positive and PEDV-negative sera were prepared in the selected serum diluent. Candidate diluents for the HRP-labeled antibody were 5% newborn bovine serum-PBST, 1% BSA-PBST, and 1% BSA-PBS. The blocking buffer, serum diluent, and antibody diluent were selected according to PI values and discrimination between positive and negative sera.

Optimization of antigen coating, blocking and serum or HRP-labeled antibody incubation time. Plates were coated with the selected antigen concentration under five conditions: overnight at 4 °C; 1 h at 37 °C followed by overnight at 4 °C; 2 h at 37 °C followed by overnight at 4 °C; 1 h at 37 °C; or 2 h at 37 °C. The blocking ELISA was then completed using the selected reagent dilutions, and the coating condition was chosen according to PI values. And then, plates coated under the selected conditions were blocked with the selected buffer for either 1 or 2 h at 37 °C. Subsequent assay steps were performed as described above, and blocking time was selected according to PI values.

To optimize serum and labeled antibody incubation time, the following combinations were evaluated: serum for 1 h and HRP-labeled antibody for 30 min; serum for 1 h and HRP-labeled antibody for 45 min; serum for 1 h and HRP-labeled antibody for 1 h; serum for 45 min and HRP-labeled antibody for 45 min; and serum for 45 min and HRP-labeled antibody for 1 h. All other procedures were performed under the selected conditions, and the incubation schedule was chosen according to PI values.

Determination of cut-off value. Characterized PEDV antibody-positive and antibody-negative sera were tested with the established blocking ELISA, and PI values were calculated. ROC analysis was performed in GraphPad Prism 5 to calculate the area under the curve and determine the cut-off. PI was calculated as PI (%) = [1 − (OD_450_ of test serum/OD_450_ of negative control)] × 100. Samples with PI values equal to or above the cutoff were classified as positive, and those below the cutoff were classified as negative.

### 2.5. Repeatability, Cross-Reactivity, and Serial-Dilution Sensitivity

Repeatability. Repeatability was evaluated with six PEDV antibody-positive and two PEDV antibody-negative sera. For intra-assay repeatability, each sample was tested five times using the same batch of S1-coated plates under identical conditions. For inter-assay repeatability, the same serum panel was tested with five independently prepared batches of coated plates. The coefficient of variation (CV) was calculated for each sample.

Cross-reactivity. Cross-reactivity was evaluated with standard sera positive for PCV2, PRV gI, PRV gB, CSFV, or PRRSV; PEDV-positive and PEDV-negative sera served as controls.

Sensitivity. Five PEDV antibody-positive serum samples were serially diluted 1:50, 1:100, 1:200, 1:400, 1:800, 1:1600, 1:3200, and 1:6400 and tested using the established blocking ELISA under the optimized assay conditions. The percentage inhibition (PI) was calculated for each dilution as described above. A dilution was considered positive when its PI value was equal to or greater than the established cutoff of 31.76%. The endpoint dilution for each serum sample was defined as the highest dilution that remained positive by the blocking ELISA. The dilutional sensitivity of the assay was evaluated on the basis of the endpoint dilutions obtained for the five sera.

### 2.6. Indirect Immunofluorescence Assay

For cell-based comparison of PEDV seroreactivity, 18 blocking-ELISA-positive, 14 negative, and 1 equivocal serum sample were examined by IFA. Briefly, Vero-81 cell monolayers were inoculated with PEDV-QY strain at a multiplicity of infection (MOI) of 0.5 and incubated at 37 °C in 5% CO_2_ for 48 h. Cells were washed twice with PBS, fixed with 50 μL/well of prechilled absolute methanol at −20 °C for 10 min, and washed three times with PBS. Serum samples were serially diluted from 1:8 to 1:256, added to the fixed cells, and incubated at 37 °C for 1 h. After three washes, FITC-conjugated goat anti-pig IgG diluted 1:250 was added and incubated at 37 °C for 45 min. Cells were washed three times, stained with 50 μL/well of bisbenzimide solution (10 μg/mL) at room temperature for 20–30 min, and washed three further times with PBS. Fluorescence was examined with an inverted fluorescence microscope. Samples showing specific green fluorescence were classified as IFA-positive; samples without specific fluorescence were classified as IFA-negative. Because PEDV-infected cells express multiple viral antigens, IFA was used as a comparison of general PEDV seroreactivity rather than as an S1-specific confirmatory assay.

### 2.7. Virus Neutralization Assay

For comparison with neutralizing antibody titers, 98 sera—72 positive, 22 negative, and 4 equivocal by blocking ELISA—were tested by virus neutralization. Vero-81 cells were seeded at 1.6 × 10^4^ cells/well and used at approximately 90% confluence. Sera were centrifuged at 12,000 r/min for 15 min, heat-inactivated at 56 °C for 30 min, and serially diluted from 1:8 to 1:256. Equal volumes of diluted serum and PEDV suspension at 0.5 MOI were mixed and incubated for 1 h at 37 °C in 5% CO_2_. The mixtures were added to washed cell monolayers and incubated for a further 1 h under the same conditions. After washing, 200 μL of maintenance medium was added per well, and the cells were incubated for 24 h. Cells were fixed with 3.7% formaldehyde for 30 min, permeabilized with 0.1% Triton X-100, blocked with 5% skim milk in PBS, and incubated sequentially with PEDV S1 monoclonal antibody and HRP-conjugated goat anti-mouse IgG. TMB was used for color development, and the reaction was stopped with 2 M H_2_SO_4_. OD_450_nm values were measured, and the neutralization inhibition rate was calculated as follows: neutralization inhibition rate (%) = [1 − (OD450 of the test sample − OD450 of the background control)/(OD450 of the virus control − OD450 of the background control)] × 100%. Based on the detection results of the same serum samples obtained by blocking ELISA and neutralization test, the coincidence rate between the two assays was analyzed.

## 3. Results

### 3.1. Expression and Identification of Recombinant S1 Protein

**Figure 1:**
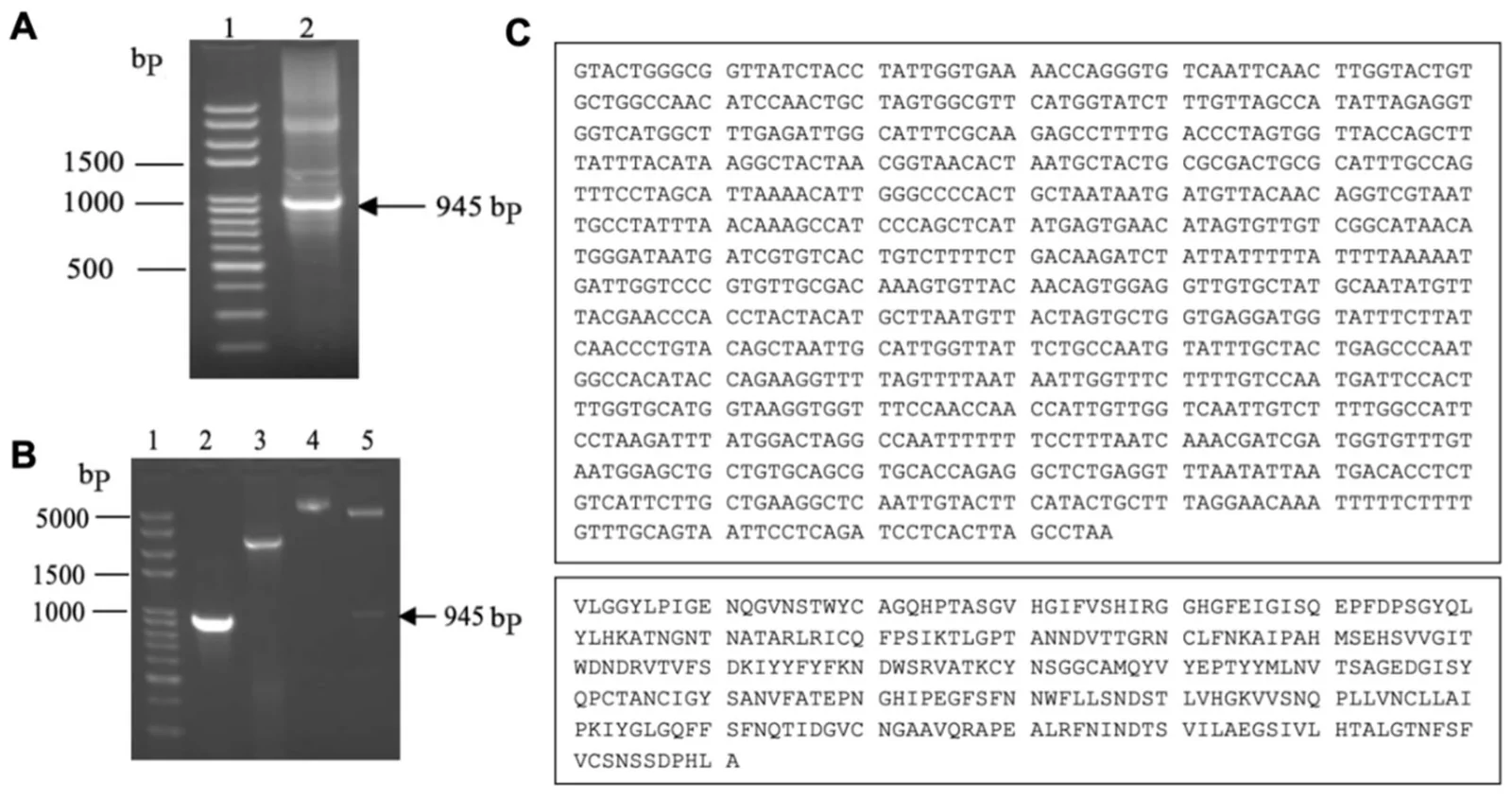
Amplification of the PEDV S1 fragment and verification of recombinant plasmid pET28a-S1. (**A**) RT-PCR amplification of the PEDV S1 target fragment. Lane 1, DNA marker; lane 2, S1 nucleotide fragment. (**B**) PCR and restriction-enzyme analysis of pET28a-S1. Lane 1, DNA marker; lane 2, recombinant-plasmid PCR product; lane 3, pET28a plasmid PCR control; lane 4, undigested pET28a-S1; lane 5, *EcoR* I/*Xho* I double-digested pET28a-S1. (**C**) Nucleotide sequence of the amplified PEDV S1 fragment and its deduced amino acid sequence.

A 945-bp fragment of the PEDV S1 gene was amplified by RT-PCR (Figure 1A). The amplicon was ligated into the pET28a(+) expression vector to generate pET28a-S1. The recombinants were confirmed by colony PCR, double-enzyme digestion (Figure 1B), and Sanger sequencing (Figure 1C).

**Figure 2:**
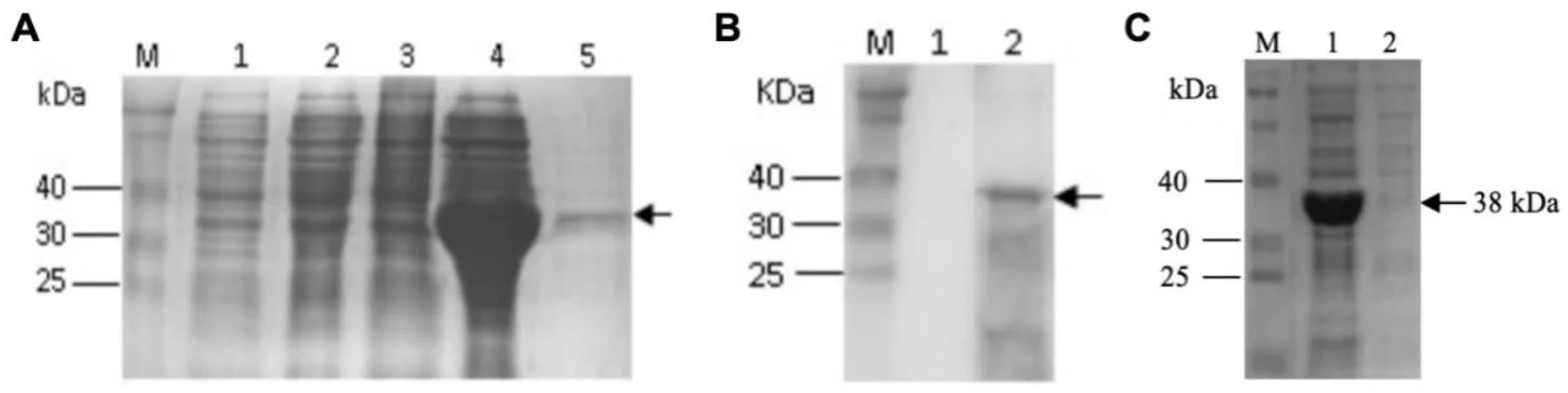
Expression and immunological characterization of recombinant PEDV S1 protein. (**A**) Expression and purification of recombinant PEDV S1 protein. M, protein marker; lane 1, pET28a/BL21 before induction; lane 2, pET28a/BL21 after induction; lane 3, pET28a-S1/BL21 before induction; lane 4, pET28a-S1/BL21 after 5 h of induction; lane 5, purified recombinant protein. (**B**) Western blot analysis. M, protein marker; lane 1, recombinant protein probed with negative serum; lane 2, recombinant protein probed with mouse anti-PEDV serum. (**C**) SDS-PAGE analysis of recombinant S1 after bacterial lysis. M, protein marker; lane 1, pellet fraction; lane 2, supernatant fraction.

Following transformation of *E. coli* BL21(DE3) with pET28a-S1, recombinant protein expression was induced with isopropyl-beta-D-thiogalactopyranoside (IPTG) at 35 °C for 5 h. SDS-PAGE revealed a prominent band of approximately 38 kDa in the induced pET28a-S1/BL21 lysate, consistent with the expected molecular mass of the truncated S1 protein (Figure 2A). Western blotting confirmed reactivity of the recombinant protein with mouse anti-PEDV S1 serum (Figure 2B). After sonication, most of the target protein was detected in the pellet rather than the supernatant (Figure 2C), indicating predominantly insoluble expression as inclusion bodies in *E. coli*. The purified antigen fraction was included in lane 5 of Figure 2A.

### 3.2. Activity of the HRP-Labeled Monoclonal Antibody

**Figure 3:**
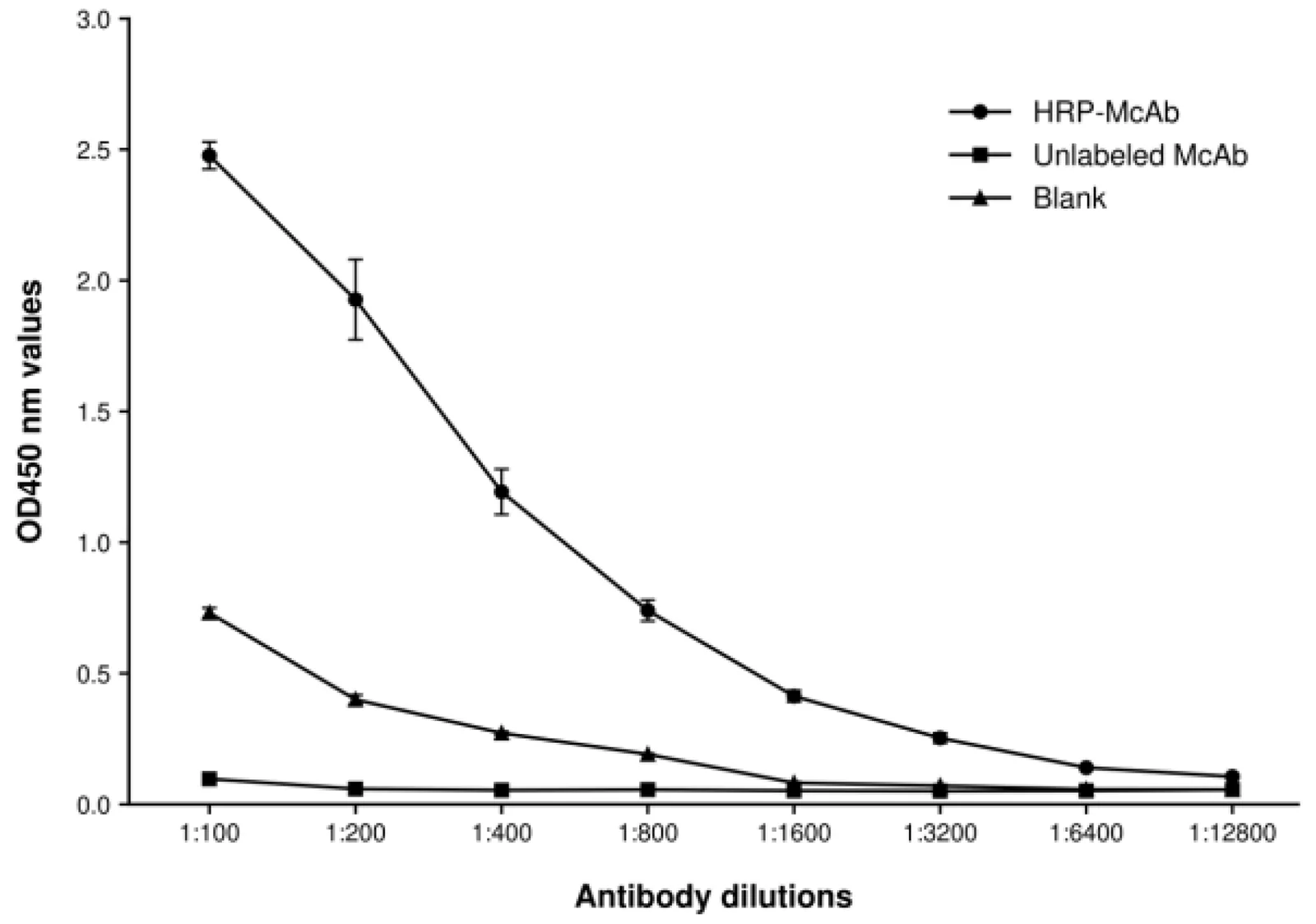
Determination of the reactive activity of the HRP-labeled S1 monoclonal antibody. Points and error bars indicate mean OD450 ± sample SD from three technical wells per condition within one experiment.

The activity and working dilution of HRP-labeled PEDV S1 monoclonal antibody was evaluated on S1-coated ELISA plates. Serial dilution produced a clear antigen-dependent signal, whereas the ascites control and the blank well control remained low, supporting successful labeling and acceptable reaction specificity. At a dilution of 1:400, the HRP-labeled antibody yielded an OD_450_ value of 1.193 ± 0.087, which was within the target working range; this dilution was therefore used for subsequent assay optimization (Figure 3). All OD450 values reported for this and the subsequent optimization experiments represent the mean ± SD of three replicate determinations.

### 3.3. Optimization of Antigen Concentration and Antibody and Serum Dilutions

**Figure 4:**
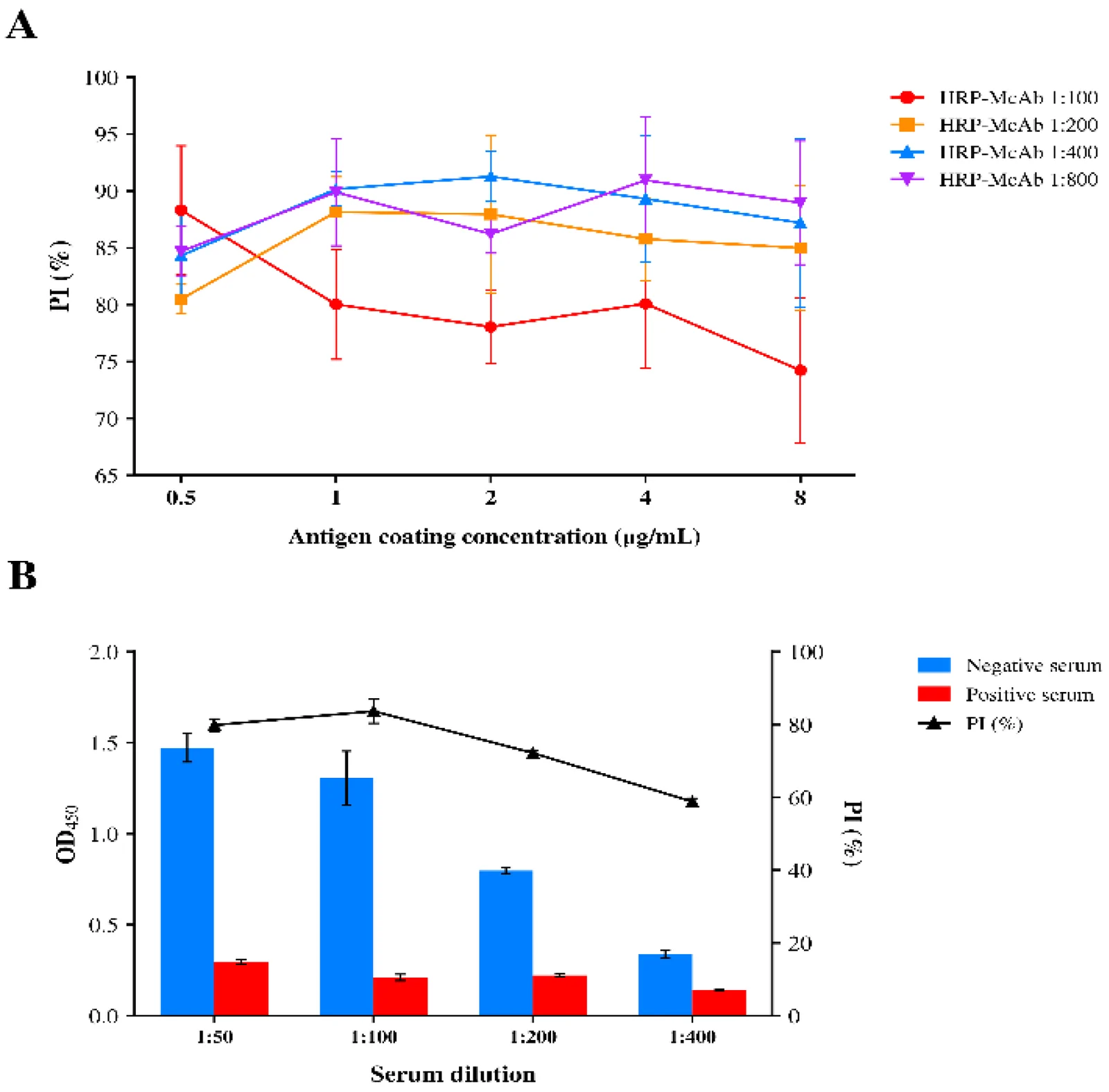
Optimization of antigen coating concentration, HRP-labeled antibody dilution, and test-serum dilution for the blocking ELISA. (**A**) Optimization of S1 antigen coating concentration and HRP-labeled antibody dilution based on percentage inhibition (PI). Each point shows the mean ± sample SD of three supplied well-level PI measurements. HRP-Ab, HRP-labeled S1 monoclonal antibody. (**B**) Optimization of test-serum dilution. Negative- and positive-serum mean OD_450_ values (bars, left axis) and mean PI (triangles, right axis), with SD error bars.

After confirming the activity of the HRP-labeled anti-PEDV S1 monoclonal antibody, the antigen coating concentration and HRP-labeled antibody dilution were further optimized in the blocking ELISA format. S1 antigen concentrations of 0.5, 1, 2, 4, and 8 μg/mL were evaluated in combination with HRP-labeled antibody dilutions of 1:100, 1:200, 1:400, and 1:800. Among the combinations tested, an S1 coating concentration of 2 μg/mL together with an HRP-labeled antibody dilution of 1:400 produced the highest PI value (91.27 ± 2.23%) and was selected for subsequent experiments (Figure 4A). Each PI value represents the mean ± SD of three replicate determinations.

Using these conditions, the test-serum dilution was subsequently optimized at 1:50, 1:100, 1:200, and 1:400. The 1:100 dilution yielded the highest PI value (83.66 ± 3.33%) while maintaining clear discrimination between PEDV-positive and PEDV-negative sera and was therefore selected as the working serum dilution (Figure 4B).

### 3.4. Optimization of Blocking Buffer and Diluents

After the antigen concentration, HRP-labeled antibody dilution, and serum dilution had been established, the blocking buffer, serum diluent, and HRP-labeled antibody diluent were evaluated in combination. Among the conditions tested, the combination of 5% newborn bovine serum in PBST for blocking and serum dilution and 1% BSA-PBS for dilution of the HRP-labeled antibody provided the greatest separation between PEDV-positive and PEDV-negative sera. Under these conditions, the negative and positive sera yielded OD450 values of 1.420 ± 0.064 and 0.135 ± 0.009, respectively, corresponding to a PI value of 90.49% (Table 1). This combination was therefore selected for subsequent assay optimization.

### 3.5. Optimization of Incubation Conditions

**Figure 5:**
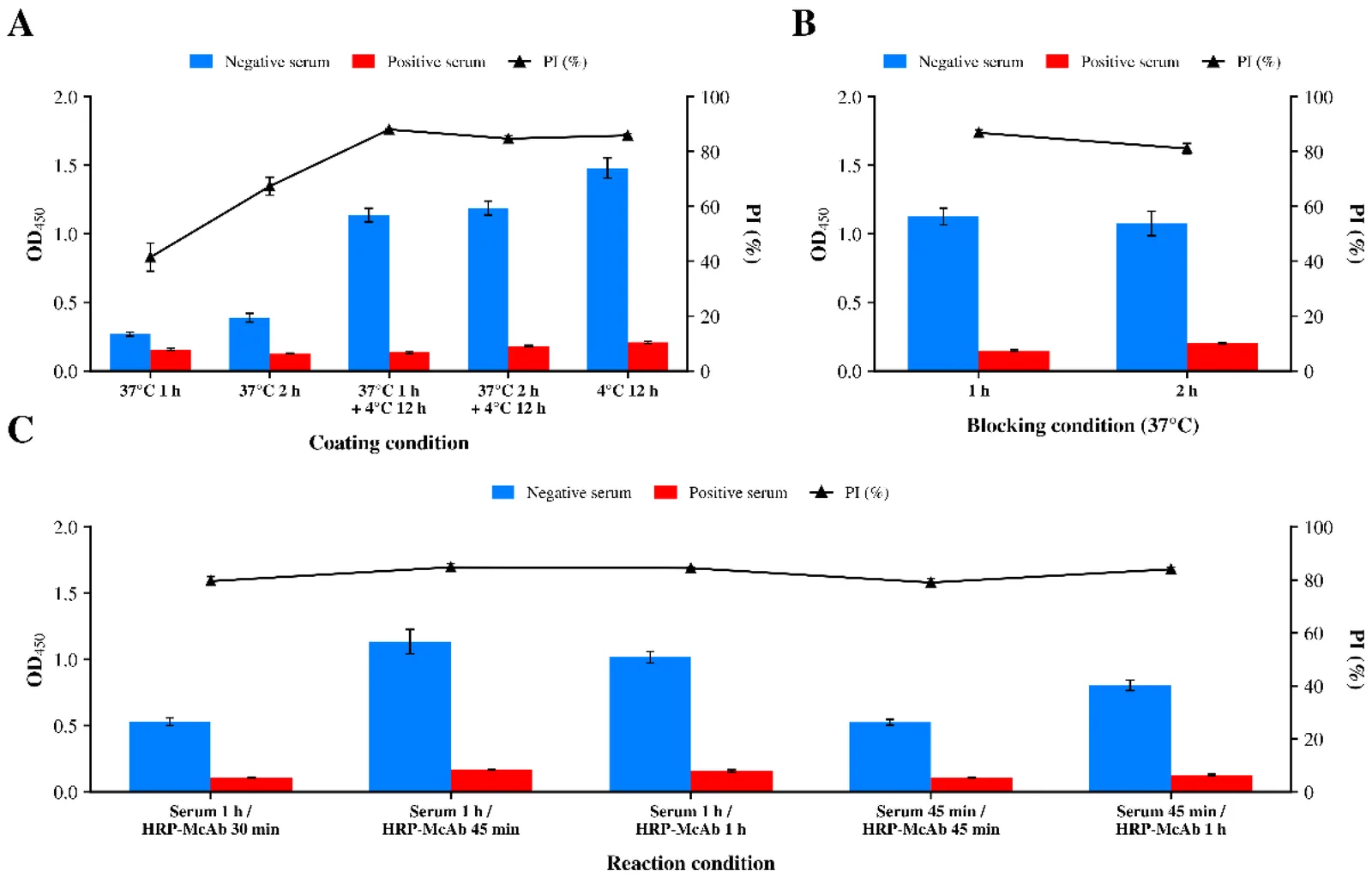
Optimization of reaction conditions for the blocking ELISA. (**A**) Antigen coating conditions. (**B**) Blocking time. (**C**) Test-serum and HRP-labeled antibody incubation times.

Five coating schedules yielded recalculated PI values of 41.48 ± 5.08%, 67.35 ± 3.25%, 88.03 ± 0.32%, 84.72 ± 1.10% and 85.85 ± 0.71% for 37 °C for 1 h, 37 °C for 2 h, and 37 °C for 1 h followed by 4 °C for 12 h, and 37 °C for 2 h followed by 4 °C for 12 h, and 4 °C for 12 h, respectively (Figure 5A). The 37 °C for 1 h plus 4 °C for 12 h schedule was retained, with PI 2.18 percentage points above the next-highest schedule, a difference of 5.66 percentage points (Figure 5B). Serum/HRP-McAb incubation schedules of 60/30, 60/45, 60/60, 45/45 and 45/60 min yielded recalculated PI values of 79.57 ± 1.71%, 84.84 ± 1.41%, 84.48 ± 0.44%, 79.05 ± 1.42% and 84.08 ± 0.78%, respectively (Figure 5C). Thus, 1 h blocking and 60/45 min serum/HRP-McAb incubation were retained as working conditions.

### 3.6. Cutoff Determination and Repeatability

**Figure 6:**
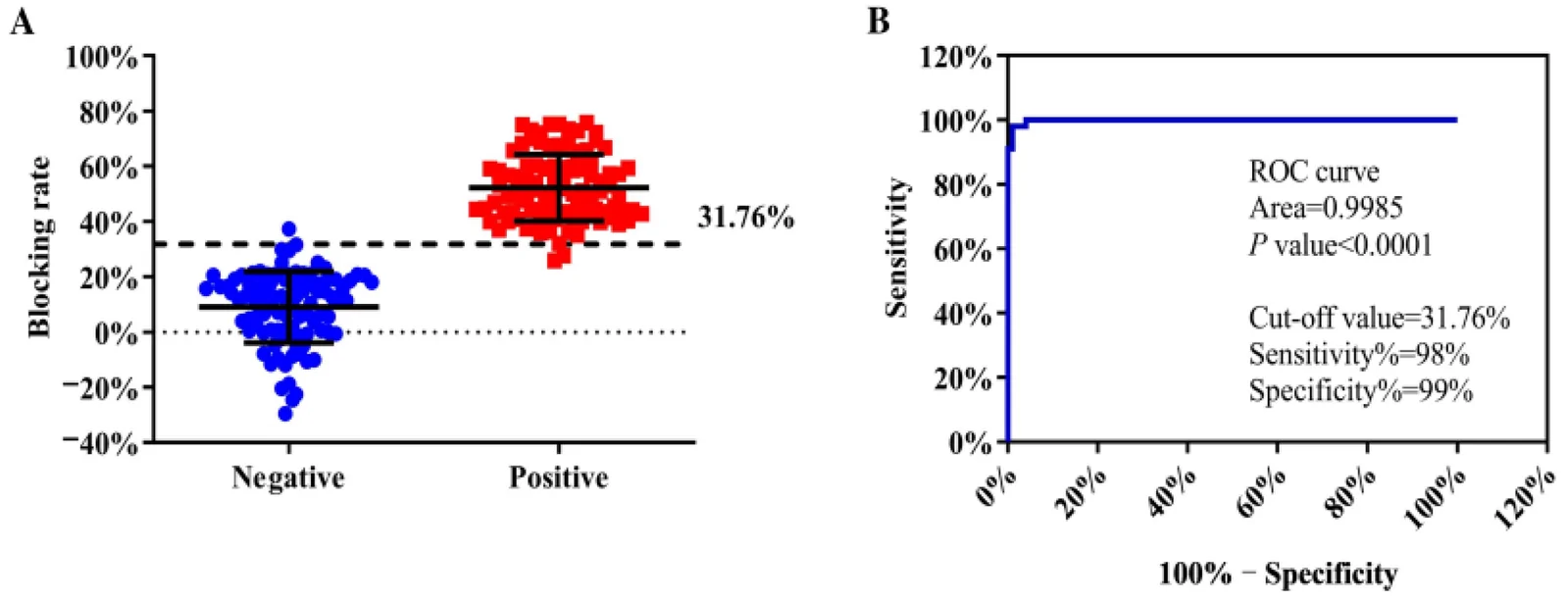
Cutoff determination and ROC analysis of the blocking ELISA. (**A**) Distribution of PI values among PEDV antibody-positive and antibody-negative sera. (**B**) ROC curve used to determine the blocking-ELISA cutoff.

**Figure 7:**
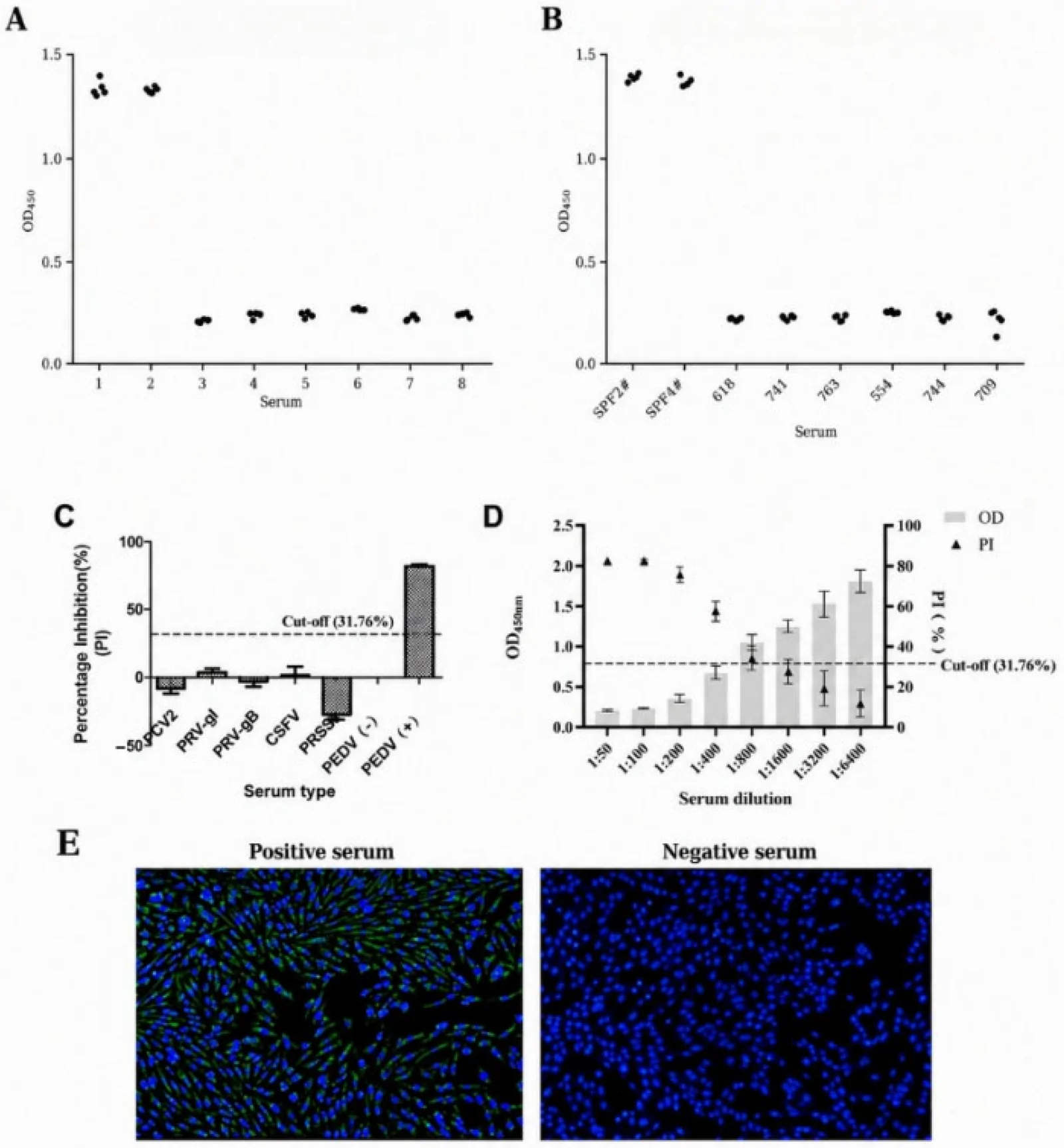
Repeatability, cross-reactivity, sensitivity and IFA comparison. (**A**) Intra-assay repeatability. Each dot represents one of five replicate measurements for each serum sample tested within the same batch. (**B**) Inter-assay repeatability. Each dot represents one measurement obtained for each serum sample across five independent batches. (**C**) Cross-reactivity with sera positive for PCV2, PRV gI, PRV gB, CSFV, or PRRSV, with PEDV-negative and PEDV-positive sera included as controls. The dashed line indicates the cutoff PI value (31.76%). (**D**) Sensitivity of five PEDV-positive sera. The sera were serially diluted and tested by the established blocking ELISA. Gray bars indicate OD450 values, triangles indicate PI (%), and the dashed line indicates the cutoff PI value (31.76%). (**E**) Representative IFA images of PEDV-infected Vero-81 cells incubated with blocking-ELISA-positive and blocking-ELISA-negative sera. Specific green fluorescence was observed with the positive serum, whereas no specific green fluorescence was observed with the negative serum.

Based on the ELISA detection results of 100 serum samples positive or negative for PEDV antibodies, a ROC curve was constructed. Testing with the established blocking ELISA yielded an area under the curve of 0.998 (*p* < 0.0001), indicating strong discrimination between the reported serum categories (Figure 6A,B). The optimal cut-off was 31.76%, at which the sensitivity and specificity were 98% and 99%, respectively. In the intra-assay repeatability test, five replicate OD450 measurements were obtained for each of six PEDV-positive and two PEDV-negative sera. The OD450 values ranged from 1.301 to 1.397 for the negative sera and from 0.205 to 0.270 for the positive sera, showing limited variation among replicates within the same assay batch (Figure 7A). The individual intra-assay CVs ranged from 0.877% to 5.605%, with a mean CV of 3.02%. In the inter-assay repeatability test, the same serum panel was evaluated across five independently prepared assay batches. The OD_450_ values ranged from 1.352 to 1.413 for the negative sera and from 0.130 to 0.259 for the positive sera (Figure 7B). The individual inter-assay CVs ranged from 0.629% to 8.753%, with a mean CV of 3.76%. All CV values were below 10%, indicating good repeatability of the established blocking ELISA.

### 3.7. Cross-Reactivity, Sensitivity, and Comparison with IFA

Cross-reactivity was assessed using sera positive for PCV2, PRV gI, PRV gB, CSFV, or PRRSV. Each non-PEDV antiserum tested negative, whereas the PEDV-positive control tested positive; no cross-reactivity was observed within this panel (Figure 7C). Sensitivity was evaluated using five PEDV-positive sera serially diluted from 1:50 to 1:6400. Four sera remained positive at dilutions up to 1:400, whereas one serum remained positive at 1:800 (Figure 7D). These results represent the dilution endpoints observed for the five sera tested.

Thirty-three clinical sera were tested by infected-cell IFA. All 18 blocking-ELISA-positive samples were IFA-positive, and all 14 blocking-ELISA-negative samples were IFA-negative (Figure 7E). Agreement among the 32 samples with determinate blocking-ELISA classifications was 32/32 (100%; 95% Wilson CI, 89.28–100%). The remaining serum had an equivocal blocking-ELISA result and was IFA-negative; it was reported separately rather than counted as concordant. Because equivocal results were present, the binary blocking-ELISA cutoff alone does not fully describe the classification procedure.

### 3.8. Agreement with Neutralizing Antibody Titer Groups

To further evaluate the clinical applicability of the established blocking ELISA and its potential correlation with host immune protection, this study systematically compared its detection results with those of the classical virus neutralization test, which measures effective protective antibodies (neutralizing antibodies). A total of 98 serum samples with known neutralizing antibody status were selected including 72 ELISA-positive, 22 ELISA-negative, and 4 equivocal samples. The consistency between the blocking ELISA results and the neutralizing antibody titers at various dilution levels (from 1:16 to ≥1:256) was systematically analyzed (detailed data are shown in Table 2).

When the serum neutralizing antibody titer was ≥1:64, the consistency rate between the blocking ELISA and the neutralization test reached 84.93% (62/73 concordant results). This threshold data indicates that the blocking ELISA method can effectively identify serum samples with high neutralizing antibody levels, and the blocking ELISA results show a dynamic positive correlation with the neutralizing antibody levels. In samples with extremely low neutralizing antibody titers (≤1:16), all blocking ELISA tests were negative, with a consistency rate of 100%, which reflects the specificity of the method in excluding non-protective samples. When the neutralizing antibody titer is moderate (1:32), the consistency rate decreased to 47.61% (10/21 concordant results), indicating that some low-titer neutralizing antibodies may not be effectively captured by this method or may be below its detection limit. When the neutralizing antibody titer increases to 1:128 and ≥1:256, the consistency rates jump to 90.91% and 100% respectively. This suggests that the blocking ELISA method is particularly excellent in detecting neutralizing antibodies with medium to high titer (≥1:64). This suggests that the blocking ELISA method is particularly effective at identifying sera with medium-to-high neutralizing antibody titers (≥1:64). This indicates that the blocking ELISA method can effectively identify serum samples with high neutralizing antibody levels, and that blocking-ELISA reactivity is positively associated with neutralizing antibody levels.

In addition to the category-level comparison, the continuous relationship between the blocking rate and neutralizing antibody titer was analyzed. Blocking PI values were correlated with serum neutralizing antibody titers (Spearman’s rank correlation, ρ = 0.484, 95% CI 0.315–0.623, *p* = 5.1 × 10^−7^; *n* = 97). These results indicate a quantitative association between blocking-ELISA reactivity and neutralizing activity.

## 4. Discussion

PEDV is a highly variable enteric RNA virus, and its prevention and control remain challenging because of persistent viral evolution and multiple transmission routes [14,19,20]. Although nucleic acid and antigen detection methods are essential for diagnosing acute infection, serological assays are indispensable for evaluating population immune status, vaccine responses, infection history, and maternal antibody transfer [16,17,18,21]. For PEDV, this is particularly important in sow herds because lactogenic immunity is a major determinant of passive protection in neonatal piglets.

In this study, a blocking ELISA based on recombinant PEDV S1 protein and an HRP-labeled S1 monoclonal antibody was established and systematically optimized. The assay showed high specificity, with no cross-reactivity against PRRSV-, CSFV-, PRV-, or PCV2-antisera. It also showed strong agreement with IFA in clinical serum testing and good repeatability, with intra-assay and inter-assay CVs of 3.02% and 3.76%, respectively. The complete agreement between the blocking ELISA and IFA further supports the reliability of the assay for detecting PEDV-reactive antibodies. However, because PEDV-infected cells express multiple viral antigens, the IFA results reflect general PEDV seroreactivity and do not independently verify exclusive detection of S1-specific antibodies. More importantly, the blocking ELISA results were associated with serum neutralizing antibody titers. Concordance with the neutralization test reached 84.93% when the neutralizing antibody titer was ≥1:64 and increased to 90.91% and 100% at titers of 1:128 and ≥1:256, respectively. These findings indicate that the assay is not only suitable for PEDV antibody screening but may also serve as a convenient indicator for identifying sera with potentially protective neutralizing activity. This positive association between blocking-ELISA reactivity and neutralizing antibody titers is consistent with previous studies reporting correlations between PEDV antibody levels and neutralizing activity [4,19]. These findings indicate that the assay is suitable for PEDV antibody screening and that its results are associated with, although they do not directly measure, neutralizing activity.

Compared with traditional indirect ELISA, the blocking ELISA format is less dependent on species-specific secondary antibodies and measures the ability of serum antibodies to compete with a defined monoclonal antibody for binding to S1 antigen. This competitive format can reduce nonspecific interference and improve analytical specificity. Existing PEDV serological methods, including S1-based indirect ELISAs and competitive/blocking ELISAs, provide useful tools for antibody surveillance [16,17,18,22]. However, they are generally used to indicate antibody exposure or total antibody levels rather than to provide an early-warning indicator linked to neutralizing antibody titers. By comparing blocking ELISA results with virus neutralization titers, this study extends the application of serological testing from simple antibody screening toward antibody monitoring that is associated with neutralizing activity [4,19,23]. Direct validation with paired maternal and piglet samples and protection-related outcomes is required before protective maternal immunity can be inferred.

Although the blocking ELISA method established in this study showed excellent performance, there are still some limitations and directions that need further improvement. (1) This method uses a single recombinant S1 protein as the detection target. Considering the high variability of PEDV S protein, especially the possible differences in the S1 region between different epidemic strains (such as classical and mutant strains), a single S1 protein may not be able to capture the complete antibody spectrum induced by all epidemic strains. This may lead to a decrease in the sensitivity of antibody detection for certain mutant strains of infection or immunity. (2) This study validated the cross reactivity of the method, but did not systematically evaluate whether the method can effectively distinguish the differences in antibody production for different genotypes or subtypes of PEDV (such as GI and GII). Nevertheless, it is reassuring that through amino acid sequence alignment, we found that the epitope recognized by the monoclonal antibody used in this study exhibits high conservation among G2 strains. This is crucial for tracing the origin of the epidemic and accurately selecting immunization vaccines. Considering the central role of neutralizing antibodies in PEDV lactogenic immunity [19] and the reported correlations between PEDV ELISA antibody levels and neutralizing activity [20], the present serum-based assay merits further validation against protection-related outcomes. Clinical validation is mainly based on serum samples with known positive and negative characteristics. In the future, it is necessary to expand the sample size and geographical sources, collecting more serum and colostrum samples from different regions, farms, and immune backgrounds (such as using different vaccine strains) for blind testing, in order to comprehensively evaluate the stability and universality of this detection method in practical and complex field application scenarios. (3) As a serological testing method, it reflects the immune status rather than acute infection. In the future, it can be combined with methods such as rapid test strips (e.g., Recombinase polymerase amplification, RPA, or Immunochromatographic Antigen Detection Test Strip) or Reverse Transcription Quantitative Polymerase Chain Reaction (RT-qPCR) for detecting PEDV nucleic acid or antigens to develop a joint detection scheme or platform that can be used for both early diagnosis of infection (antigen/nucleic acid detection) and later immune monitoring (antibody detection), in order to provide more comprehensive disease management information.

Overall, the PEDV S1-based blocking ELISA provided reproducible antibody detection within the serum panels evaluated. Its category-specific agreement with higher neutralizing antibody titers supports further investigation as an immune-monitoring assay. Direct validation with defined maternal and piglet samples and protection-related outcomes is required before the method can be used to infer maternal antibody transfer or passive protection. Future studies should also include multicenter validation in larger clinical cohorts at various specific antibody levels.

## 5. Conclusions

This study successfully established a highly efficient and specific blocking ELISA method for detecting PEDV antibodies. This method utilizes recombinant PEDV S1 protein as the coating antigen and HRP-labeled S1-monoclonal antibodies as the detection probe. Through systematic optimization of the reaction system, the optimal assay conditions were established, and the blocking ELISA showed 100% concordance with the indirect immunofluorescence assay used as a reference serological method in this study. It exhibits strong specificity, showing no cross-reactivity with the sera against other common swine viruses, along with a stable reproducibility. Furthermore, there is a statistically significant correlation between the serum IgG antibody levels detected by this method and the serum neutralizing antibody titers reflecting the actual protective immunity of the body. This indicates that the blocking ELISA established in this study is not only an effective tool for rapid screening of PEDV infection but also has the potential to serve as a convenient and reliable “indicator of immune protection efficacy”. Therefore, this method is suitable for large-scale epidemiological surveillance, evaluation of immune effects after vaccination, providing a powerful technical support for timely understanding of the immune status of swine herds, optimizing immunization programs, and formulating effective PEDV prevention and control strategies.

## Figures and Tables

**Table 1:** Determination of the Optimal Blocking Buffer and Antibody Diluent.

Serum Diluent	Serum Type	Blocking Buffer 1		Blocking Buffer 2	
		Diluent 3 (HRP-McAb)	Diluent 4 (HRP-McAb)	Diluent 3 (HRP-McAb)	Diluent 4 (HRP-McAb)
1	Negative	0.533 ± 0.015	1.420 ± 0.064	**0.442 ± 0.016**	0.653 ± 0.052
	Positive	0.109 ± 0.006	0.135 ± 0.009	**0.132 ± 0.002**	0.169 ± 0.003
2	Negative	0.437 ± 0.006	0.434 ± 0.023	0.393 ± 0.024	0.246 ± 0.005
	Positive	0.216 ± 0.008	0.094 ± 0.004	0.133 ± 0.006	0.090 ± 0.002
3	Negative	0.116 ± 0.003	0.218 ± 0.008	0.101 ± 0.002	0.052 ± 0.003
	Positive	0.107 ± 0.009	0.117 ± 0.002	0.088 ± 0.002	0.052 ± 0.005
4	Negative	0.162 ± 0.004	0.247 ± 0.004	0.136 ± 0.004	0.140 ± 0.004
	Positive	0.103 ± 0.010	0.062 ± 0.006	0.058 ± 0.002	0.057 ± 0.003

Note: Blocking buffer 1, PBST containing 5% newborn bovine serum; blocking buffer 2, PBST containing 1% BSA; diluent 1, same as blocking buffer 1; diluent 2, same as blocking buffer 2; diluent 3, PBS containing 5% newborn bovine serum; diluent 4, PBS containing 1% BSA. HRP-Ab, HRP-labeled S1 monoclonal antibody. Bold values indicate the selected optimal condition.

**Table 2:** Categorical concordance between the blocking ELISA and virus neutralization test.

Neutralization Titer	Total Number of Samples	Blocking ELISA			Concordance (%)
		Positive	Negative	Equivocal	
≥1:256	3	3	0	0	100
1:128	22	20	1	1	90.91
1:64	48	39	8	1	81.25
1:32	21	10	9	2	47.61
≤1:16	4	0	4	0	100

## Data Availability

No original code is reported in this study. Information required to reanalyze the data is available from the corresponding author upon reasonable request.

## Abbreviations

The following abbreviations are used in this manuscript:

BSA	Bovine serum albumin
CSFV	Classical swine fever virus
CV	Coefficient of variation
DAB	3,3′-Diaminobenzidine
ELISA	Enzyme-linked immunosorbent assay
FITC	Fluorescein isothiocyanate
HRP	Horseradish peroxidase
HRP-McAb	Horseradish peroxidase monoclonal antibody
IFA	Indirect immunofluorescence assay
IPTG	Isopropyl-β-D-thiogalactopyranoside
MOI	Multiplicity of infection
OD	Optical density
NBS	Newborn bovine serum
PBS	Phosphate-buffered saline
PBST	Phosphate-buffered saline containing Tween 20
PCV2	Porcine circovirus type 2
PED	Porcine epidemic diarrhea
PEDV	Porcine epidemic diarrhea virus
PI	Percentage inhibition
PRRSV	Porcine reproductive and respiratory syndrome virus
PRV	Pseudorabies virus
PVDF	Polyvinylidene difluoride
ROC	Receiver operating characteristic
RT-PCR	Reverse transcription polymerase chain reaction
RVA	Rotavirus A
SDS-PAGE	Sodium dodecyl sulfate polyacrylamide gel electrophoresis
TGEV	Transmissible gastroenteritis virus
TMB	3,3′,5,5′-Tetramethylbenzidine
